# Assessment of Knowledge and attitudes of psychiatric nurses toward electroconvulsive therapy

**DOI:** 10.1192/j.eurpsy.2022.1578

**Published:** 2022-09-01

**Authors:** N. Herch, R. Lansari, Y. Bennasr, W. Melki

**Affiliations:** 1 Razi Psychiatric Hospital, Psychiatry D, Manouba, Tunisia; 2 Razi Hospital, Psychiatry D, Tunis, Tunisia; 3 Razi Psychiatric Hospital, Psychiatry D, Nabeul, Tunisia

**Keywords:** Nurses, attitude, Electroconvulsive therapy, knowledge

## Abstract

**Introduction:**

knowledge and atittudes of psychiatric nurses should be continuously evaluated and updated to ensure a quality care in electroconvulsive therapy (ECT) unit.

**Objectives:**

Assessment of the psychiatric nurses knowledge and attitudes towards ECT.

**Methods:**

A cross sectional descriptive study was conducted in multiples psychiatric departments in Razi Hospital Tunisia between January and April 2021.We asked 30 psychiatric nurses using a questionnaire evaluating their knowledge and attitudes towards ECT technique and its impact on the medical care.

**Results:**

Our study revealed a lack of knowledge on ECT among psychiatric nurses.In fact, 93% of nurses reported that schizophrenia represents the most frequent indication. Pregnancy was considered as a contraindication by all participants. ECT was not recognized as a first line treatment and Only few knew the complet medical checkup before ECT.As for their opinion about this technique, 73 % of the nurses have a positive attitude towards ECT and think that it is very effective .

**Conclusions:**

Nurses have a major role in the progress of every ECT-session .For that, specific training can only improve their knowledge and promote more positive attitude toward ECT.

**Disclosure:**

No significant relationships.

